# Attentional Differences Between Males and Females for Emojis During the Emotional Stroop Task

**DOI:** 10.7759/cureus.60428

**Published:** 2024-05-16

**Authors:** Deeksha Patel, Om Lata Bhagat, Abhinav Dixit

**Affiliations:** 1 Physiology, All India Institute of Medical Sciences, New Delhi, Delhi, IND; 2 Physiology, All India Institute of Medical Sciences, Jodhpur, Jodhpur, IND

**Keywords:** emoji, reaction time, attention, emotion, emotional stroop task

## Abstract

Introduction

Cognitive load can be intensified by emotional components such as emotive words or facial expressions. Sex differences influence both emotional and cognitive functions for emotional facial expressions. Emojis, in contrast to human faces, serve as digital cues conveying emotional nuances in communication. The present study aimed to compare attentional differences prompted by emojis.

Methods

This study aimed to compare attentional differences in males and females elicited by emojis in 100 healthy adults (50 males and 50 females) within the age group of 18 to 40 years (mean ± SD: 27.87 ± 5.37 years) while performing the emotional Stroop task (EST). The EST comprised emojis depicting four emotions (happy, fear, sad, and angry) and emotionally charged words conveying similar emotions. An independent sample t-test was used to compare the reaction times among males and females.

Results

Results showed males had significantly longer reaction times than females across all task conditions. Both genders exhibited significant differences in reaction times across task conditions, except inhibition. Overall, reaction times increased notably from neutral to incongruent conditions for both genders. This suggests that response times increased significantly from neutral to incongruent conditions.

Conclusion

Emojis introduced in the EST revealed gender-related differences in attentional processing. This study showed the greater proficiency of females in emotional processing during EST compared to males. These findings contribute to our understanding of how gender is associated with cognitive responses to emotional stimuli in digital communication contexts.

## Introduction

Adequate integration of information from the external world and behavioral adaption according to the situation for healthy execution of the body can be achieved by various interactions in the brain. One such interaction is that of emotion and cognition. The response of individuals during emotional situations also impacts their cognition and behavior [1]. Similarly, cognition also affects emotional processing. Therefore, a proper balance of cognition and emotion is necessary for the functioning of everyday life [2,3]. The interference produced by varying the degree of cognitive load can be quantified by the Stroop task [4]. The exact neuronal mechanism of emotion-cognition interaction is still elusive but some neuroimaging studies have indicated different brain areas involved in such interactions [5,6]. The brain areas activated in response to emotional stimuli are the anterior cingulate cortex (ACC), amygdala, insula, limbic area, and prefrontal cortex [7]. Whereas the areas activated during Stroop task performance are the insula, ACC, prefrontal cortex, and superior temporal lobe [8,9]. The activation of common brain areas suggests the association of emotional processing with cognitive control and vice-versa.

The Stroop task is a neuropsychological task that assesses the cognitive control of an individual [10]. The emotional Stroop task (EST) is an extensively used task to measure attention predisposition for different prime emotions [11,12]. It is the modified version of J.R. Stroop’s (1935) classical color-word Stroop task [13]. Unlike classical Stroop tasks which have ink color and the name of the color, EST uses emotional words of different valence instead of the name of the color. In the EST, the emotional (happy, sad, anger, and fear) and neutral (chair, pen, cup) words are written in different colors, and subjects are asked to identify the color ignoring the emotional word [11]. It took longer for the subject to identify the ink color with emotional words than the neutral word. This delay in response due to emotional words is known as the emotional Stoop effect (ESE) [14].

There are several mechanisms to explain the ESE. Emotional words capture the attention of the subject and cause delays in response. This is one mechanism explaining the ESE. Response time may vary individually because of different attention orientations captured by the emotional stimulus. This effect may be influenced by environmental factors, mood, anxiety traits, or psychological disorders [15]. The other mechanism is the temporary freezing mechanism which explains the effectiveness of the brain to store the previous threat or pleased information from the surroundings. When these emotions presented during a task capture momentary attention it causes a delayed response [16].

EST is being extensively used for measuring response time in healthy as well as patient populations for emotional stimuli. Various modifications in EST have been developed in neuropsychological studies to assess the attention in different groups of individuals based on age and gender. Word-Face EST is one of the modifications in which emotional faces are superimposed with similar or contradictory emotional words and the subject has to focus on emotional faces while ignoring the words [17].

In the growing era of digital communication, emojis play a key role in adding the emotional essence to messages. Previous studies also suggested the neurological correlations of emojis with human faces [18,19]. Males and females due to their different anatomical and physiological orientations react differently to different kinds of emotional exposures [20,21]. However, the emotional dissent in males and females for various expressions of emojis is still vague. So, this study was designed to assess the attentional difference among males and females during EST including four different valences of emojis (happy, sad, fear, and angry) each superimposed with an emotional word (happy, sad, fear, and angry).

## Materials and methods

This study was conducted in the Cognitive Neurophysiology Laboratory, Department of Physiology, All India Institute of Medical Sciences (AIIMS), Jodhpur, Jodhpur, India. It was approved by the Institutional Ethics Committee of AIIMS, Jodhpur with reference no. AIIMS/IEC/2018/796 and written informed consent was taken from participants. The comparative study includes a total of 100 healthy participants, randomly sampled into 50 males (age: mean ± SD: 29.38 ± 5.771 years) and 50 females (age: mean ± SD: 25.06 ± 4.288 years). They were included based on education (minimum five years of schooling), familiarity with the use of the computer, and without any history of neurological disease/head trauma, drugs or alcohol abuse, or consumption of sedatives in the past three days. The psychological general well-being index (PGWBI) was used to assess the emotional stability of individuals [22]. The raw index score (RIS) and the PGWBI score for six domains (i.e., anxiety, depression, positive well-being, self-control, general health, and vitality) were calculated separately. The participants having RIS of more than or equal to 73 out of 110 were considered emotionally stable and included in the study. The average score or RIS of the participants was calculated at 96.97.

**Figure 1:**
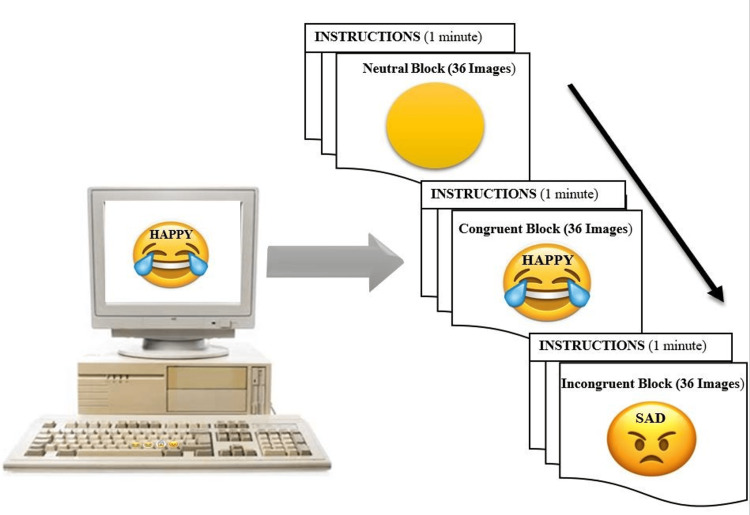
Graphical representation of emotional Stroop task

The subjects were assessed on a computerized Emoji-Word EST that was designed using Superlab 5 software (Cedrus Corporation, Los Angeles, United States). As seen in the traditional version of the color-word Stroop task, the EST also consisted of three blocks (neutral, congruent, and incongruent). The selections of emojis were based on “emoji sentiment score” and “emoji sentiment ranking” [23]. In the neutral condition block “emojis without any facial expression and words” were used. In the congruent condition block of EST “emojis having emotional expressions (happy, sad, fear and angry)” were superimposed with the same emotional words as indicated by the emotion of emojis. Unlike the congruent condition block, in the incongruent condition block of EST, “emojis having emotional expressions (happy, sad, fear and angry)” were superimposed with words of different valence. The emotional words (happy, sad, fearful, and angry) did not correspond to the emotion represented by emojis. The subjects were required to respond to the emotion of the emoji and not the emotional word itself. Participants were asked to respond by pressing the 4 assigned keys on the keyboard that represent particular emotions of emoji for each three blocks. The stickers of particular emotions have already been pasted on assigned keys. The subjects responded to emotions while ignoring the emotional words. Each block consisted of 36 images. The representative task design is shown in Figure 1.

The reaction time for each block in all three conditions was recorded for each subject. The facilitation, inhibition, and Stroop effect (SE) were calculated for all the subjects based on their reaction times. Facilitation was calculated by subtracting the reaction time of the congruent block from the neutral condition for each trial. Inhibition was calculated by subtracting the reaction time of the incongruent condition from the neutral condition. Finally, the SE was calculated by subtracting the reaction time of the incongruent block from the reaction time of the congruent block. The facilitation, inhibition, and SE of males and females during the EST were then compared.

Statistical analysis

The comparison between groups was calculated by using an independent sample t-test for males and females. Repeated measures analysis of variance (ANOVA) was used to make within-group comparisons of neutral, congruent, and incongruent groups in both males and females. The results were calculated using IBM SPSS Statistics for Windows, Version 25 (Released 2017; IBM Corp., Armonk, New York, United States).

## Results

In this study, participants were recruited to evaluate their performance on the Emoji-Word EST. The mean age of participants was 27.87 ± 5.37 years. A PGWBI questionnaire was used to assess the emotional stability and general well-being of participants. 

Before conducting the analysis, the assumption for normally distributed differences scores were considered satisfied as the skewness and kurtosis levels were less than the maximum allowable values for a t-test (i.e., skew < |2.0| and kurtosis < |9.0|: Posten 1984). An independent sample t-test was used to make a comparison in reaction time for males and females. The results indicated that reaction time in males was greater as compared to the females for all three blocks i.e. neutral (t(3.256) = 98, p < 0.05), congruent (t(10.74) = 98, p < 0.05) and incongruent (t(12.71) = 98, p < 0.05). In addition, increased reaction time in males as compared to females was also observed in facilitation (t(6.481) = 98, p < 0.05), inhibition (t(1.710) = 98, p > 0.05), and SE (t(2.387) = 98, p < 0.05) (Table 1).

The repeated measure ANOVA was used for within-group comparison in both males and females. The results indicated that the mean time of participants differed significantly in all conditions in both males and females. For females, F (2,49) = 347.4, p < 0.001 for the EST, and for males, F (2,49) = 322.6, p < 0.001 for the EST. The post hoc tests using Bonferroni correction revealed the male and female participants differed significantly (p < 0.001) in all conditions except for facilitation and SE pair. These results suggested that the time taken by participants to respond to pictures increased significantly from neutral to incongruent conditions (Table 1).

**Table 1:** Comparison of reaction times between males and females for neutral block, congruent block, and incongruent block A p-value ≤ 0.05 was considered as significant.

Conditions	Sex	Reaction time (millisecond) mean and SD		t	df	Significant (2- tailed)
Neutral	M	448.5	162.2	3.256	98	0.0286
	F	359.3	105.7			
Congruent	M	1314	184.3	10.74	98	0.0013
	F	987.2	111.6			
Incongruent	M	1539	187.2	12.71	98	0.0252
	F	1162	95.42			
Facilitation	M	865.8	221.9	6.481	98	0.0007
	F	628	134.4			
Inhibition	M	851.4	293.1	1.710	98	0.0905
	F	758.7	246.7			
Stroop effect	M	223.8	108.3	2.387	98	0.0432
	F	175.8	91.97			

## Discussion

In the present study response time was assessed for both males and females. There was a gradual and significant increase (p < 0.001) in reaction time from neutral to congruent to incongruent block; the average value in an incongruent block was found to be maximum. The results of the EST were consistent with that of the classical color-word Stroop task. Participants took the least time to respond to neutral block images and the maximum time to respond to incongruent block images. These results indicated interference for the incongruent block images. The neutral block does not contain any emotional emojis so processing for the stimuli might be faster in brain areas compared to the incongruent block which contains emotional emojis. The SE was also observed, which was indicative of automatic reading of the written word during attention control toward emotional emojis.

Males showed a significantly higher level of interference than females in recognizing the expressions of emojis. The possible reason for this difference could be the stronger arousal of females for emotional stimuli. Females have greater expressivity and higher arousal for the emotional stimuli which could be responsible for their faster response [24].

Females showed more facilitation and less inhibition than males during the Stroop task. Females also had a lesser SE than males which indicated that the females had greater attentional control and better ability to inhibit inappropriate responses during the task. These findings were similar to those reported by Mekarski et al., the color-word Stroop task was used to compare the response time in males and females. The response time to respond for the word, as well as color, was found to be less for females than males. They used a color-word Stroop task having congruent and incongruent images. They suggested that more errors occurred during the processing of words than colors. This is possibly due to the morphological transformation of letters which might impart meaning at the cortical level [25]. Wang et al. also observed similar findings during the face-word Stroop task and subsequent memory task. Males and females were separately assessed during the face-word Stroop task and subsequent memory task and results showed that males had better facial recognition. In contrast, females were more efficient during memory tasks during emotional conflict. They also observed that females memorized incongruent faces more easily than males. This could be a possible reason for the better performance of females during conflicting situations [26].

Gender differences can be seen in the processing of different emotional stimuli. Isaac et al. noticed greater interference for angry and threatening faces as compared to neutral and happy faces in sad moods during the EST which suggests attentional interference could be more in mood-congruent emotions [27]. The effect of positive emotions was explained by Liu et al. who suggested the quick and automatic detection of positive emotion irrespective of gender [28]. Herrmann et al. during an fMRI study observed a stronger activation of the amygdala, prefrontal cortex, occipitotemporal cortex, and thalamus during the processing of angry and fearful faces in females than in males [29]. The greater activation of brain areas involved in emotional processing indicates the greater and faster response of females during this emotional valence compared to males. Saylik et al. found that females had faster response times in recognition of both positive and negative faces as compared to males for emotion recognition tasks, having a series of emotional faces as well as working memory tasks [30].

In summary, our research indicated that females exhibited a faster response to emotional emojis than males when presented with an EST.

Strengths

The study's findings are consistent with existing research on gender differences in emotional processing and attentional control tasks, which enhances the credibility of the results. This study replicated the color-word Stroop task and obtained similar results, adding to the scientific validity of the study. The research adds to the conversation on gender disparities in cognitive functions, specifically about emotional stimuli and attentional control.

Limitations

The study specifically examines differences in response time in an EST. There is no comparison of response time in non-emotional digital content among males and females. Results may vary when looking at other cognitive tasks, emotional stimuli, and non-emotional stimuli. Other factors such as personality traits, life experiences, and hormonal variances may also play a role.

Implications

Gender differences in emotional processing and attentional control could impact clinical psychology and psychiatry. Tailored interventions could be created to address conditions like anxiety disorders or mood disorders based on these differences.

## Conclusions

The results of the present study showed that emotional responses elicited by emojis have an impact on attention and emotional experience in both genders. However female participants showed lesser response time, interference, inhibition, and SE than males while encountering emotional stimuli during EST. Moreover, the facilitation was higher in females compared to males. Therefore, it was concluded that females have greater attentional control, expressivity, and recognition of incongruent block images.

## Figures and Tables

**Figure 2:**
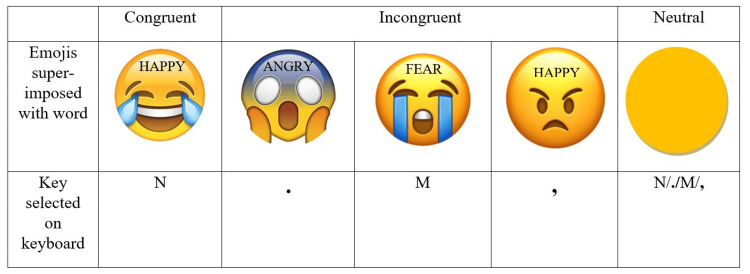
Sequences of stimuli presented during emotional Stroop task
